# Effect of Capsaicin and Other Thermo-TRP Agonists on Thermoregulatory Processes in the American Cockroach

**DOI:** 10.3390/molecules23123360

**Published:** 2018-12-18

**Authors:** Justyna Maliszewska, Milena Jankowska, Hanna Kletkiewicz, Maria Stankiewicz, Justyna Rogalska

**Affiliations:** 1 Department of Animal Physiology, Faculty of Biology and Environmental Protection, Nicolaus Copernicus University, 87-100 Toruń, Poland; kletkiewicz@umk.pl (H.K.); rogal@umk.pl (J.R.); 2 Department of Biophysics, Faculty of Biology and Environmental Protection, Nicolaus Copernicus University, 87-100 Toruń, Poland; mjank@umk.pl (M.J.); stankiew@umk.pl (M.S.)

**Keywords:** temperature perception, TRP receptors, behavioral thermoregulation, American cockroach, allyl isothiocyanate, capsaicin, camphor, menthol, thymol

## Abstract

Capsaicin is known to activate heat receptor TRPV1 and induce changes in thermoregulatory processes of mammals. However, the mechanism by which capsaicin induces thermoregulatory responses in invertebrates is unknown. Insect thermoreceptors belong to the TRP receptors family, and are known to be activated not only by temperature, but also by other stimuli. In the following study, we evaluated the effects of different ligands that have been shown to activate (allyl isothiocyanate) or inhibit (camphor) heat receptors, as well as, activate (camphor) or inhibit (menthol and thymol) cold receptors in insects. Moreover, we decided to determine the effect of agonist (capsaicin) and antagonist (capsazepine) of mammalian heat receptor on the American cockroach’s thermoregulatory processes. We observed that capsaicin induced the decrease of the head temperature of immobilized cockroaches. Moreover, the examined ligands induced preference for colder environments, when insects were allowed to choose the ambient temperature. Camphor exposure resulted in a preference for warm environments, but the changes in body temperature were not observed. The results suggest that capsaicin acts on the heat receptor in cockroaches and that TRP receptors are involved in cockroaches’ thermosensation.

## 1. Introduction

Capsaicin is known to induce changes in thermoregulatory processes of animals. In mammals, this alkaloid has been shown to induce hypothermia that occurs both at physiological (skin vasodilation, suppression of thermogenesis and salivation) and behavioral (cold-seeking behavior) response levels [1,2,3,4]. Some reports have shown the effect of capsaicin on thermoregulatory processes in invertebrates such as an induced preference for lower temperatures in *Periplaneta americana* [5] or *Leptinotarsa decemlineata* [6]. Moreover, exposure to this alkaloid has been reported to change the responsiveness to noxious heat in the American cockroach [7] and in land snail *Megalobulimus abbreviatus* [8]. Nocifensive behavioral responses after capsaicin application have also been observed in the medicinal leech [9]. However, the mechanism by which capsaicin induces thermoregulatory responses in invertebrates has not been elucidated yet.

Insects are ectotherms, and therefore their body temperature depend nearly directly on ambient thermal conditions. They regulate their body temperature mainly by behavioral mechanisms, but some physiological contributions to body temperature adjustment are also observed, such as warm-up using flight muscles [10]. A well-known ability of insects to regulate their body temperature (mostly behaviorally, but also endogenously) indicates that they possess structures that detect exterior and interior temperatures. Insect thermoreceptors are located both inside the body as well as peripherally. It has been shown that in *Periplaneta americana* central thermoreceptors are located in thoracic ganglia [11]. Hamada et al. [12] showed the existence of the thermoreceptors essential for selection of preferred temperature in *Drosophila*’s brain. Insects’ peripheral thermoreceptors are located on antennae and tarsi. Receptors that respond to cold are mostly located on antennae, together with hygroreceptors, which have been found in bees, locusts, cockroaches, stick insects, mosquitoes (*Aedes*) and crickets [13]. Recent research has shown that there are two types of thermoreceptors on insects’ antennae—One which responds to warm and one which responds to cold ambient temperatures. These structures are highly sensitive even to small temperature changes (below 0.5 °C) [14].

Some members of the TRP family are involved in temperature sensation and they are named thermo-TRP. It has been demonstrated that insect thermoreceptors belong to the TRP family. The first member of this family discovered to participate in thermosensation was a TRPA subfamily member named Painless. This receptor, which is located both in the central and peripheral *Drosophila* nervous system is responsible for detecting noxious heat. Mutants deprived of Painless showed diminished reactions to high ambient temperatures (42 °C). The second receptor responsible for avoidance of noxious heat in insects is Pyrexia. Flies deprived of this receptor were paralyzed faster during the exposure to 40 °C. Pyrexia is expressed in sensory neurons that innervate bristles located on the dorsal part of the thorax, antennae and proboscis. Another insect warm-activated receptor is dTRPA1, which opens at 25–27 °C. dTRPA1 participates in thermal preferences optimal for growth in *Drosophila* larvae [15,16]. Insect orders differ in terms of TRP channels presence. In Coleoptera and Hymenoptera, another member of TRPA subfamily has been found, although not mentioned before TRPA5. This is absent in *Drosophila* but its role in the perception of temperature is unknown. Moreover, dTRPA1 is absent in Hymenoptera, but another channel—HsTRPA (Hymenoptera specific TRPA) has been discovered in this order [17]. It is suggested that HsTRPA in Hymenoptera is the equivalent of dTRPA1, and it functions as a detector of an increase in the nest temperature (>36 °C) inducing nest-cooling behavior [18]. So far, the knowledge about insect structures involved in the perception of cold has been limited. In *Drosophila*, two members of the TRPC subfamily, TRP and TRPL, are required for cool avoidance [19]. It is also suggested that another receptors involved in cold perception in flies might be TRPP subfamily members, brivido-1, -2, and -3. Gallio et al. [8] showed that neurons expressing brivido in the second antennal segment selectively and rapidly respond to cooling and are not activated by hot stimuli. Kwon et al. [20] demonstrated that a member of the TRPV subfamily, Inactive, may be involved in cold sensation in *Drosophila* larvae determining the avoidance of cool temperatures. Three other TRP members were proposed to be involved in response to noxious cold (<10 °C): Nompc, Trpm and Pkd2 [21].

In vitro research has shown that insect thermo-TRP are activated not only by temperature but also by various natural substances (as shown in Table 1). Insect warmth receptors (Painless and dTRPA1) are activated by allyl-isothiocyanate (AITC; which gives horseradish the pungent taste), whereas HsTRPA, specific for Hymenoptera species, is activated by camphor and inhibited by menthol. Insect cold receptor TRPL is inhibited by menthol and thymol, the component of common thyme. Camphor activates *Drosophila* cold receptor TRPL and inhibits heat receptor Painless. However, there are limited data concerning the thermoregulatory behavior of insects after the application of agonists or antagonists of thermo-TRP. Kohno et al. [18] demonstrated that the injection of menthol, which suppresses the activity of HsTRPA, induces bees preference for warmer regions. Zermoglio et al. [22], Olszewska and Tęgowska [23] and Maliszewska and Tęgowska [5] studied the effect of capsaicin and capsazepine on insects’ thermal preferences and their results suggest the existence of structures sensitive to these substances. Detailed analysis of the effect of selected ligands on thermoregulatory processes is presented in Table 2.

The aim of our study was to evaluate the effect of TRP ligands on the American cockroach’s thermoregulation. The body temperature at constant ambient temperature, as well as their influence on the cockroach’s thermal preferences in a wide thermal range. We studied the effect of various ligands that have been shown to activate (allyl isothiocyanate) or inhibit (camphor) warm receptors, as well as activate (camphor) or inhibit (menthol and thymol) cold receptors in insects. In our previous studies [5,7], we demonstrated changes in thermal preferences in cockroaches exposed to capsaicin and capsazepine; thus, the present study was aimed at determining only the body temperature changes after exposure to these ligands. This let us observe whether tested TRP ligands change physiological thermoregulation. As far as we are concerned, this is the first mention of insects’ body temperature changes after exposure to capsaicin and other TRP-ligands. It is of great importance for understanding thermoregulation in insects.

## 2. Results

### 2.1. Behavioral Thermoregulation of Cockroaches Exposed to TRP Ligands

Ambient temperature preferred by American cockroaches was influenced by thermo-TRP ligands: camphor, allyl isothiocyanate (AITC), menthol and thymol (d.f. = 3, F = 140.438, *p* < 0.001), but the effect was not significantly different when analyzed for various concentrations of the ligands (d.f. = 4, F = 1.457, *p* = 0.213). Furthermore, significant interaction between the substance and its concentration was observed (d.f. = 12, F = 8.106, *p* < 0.001). Post hoc tests revealed significant differences between camphor and AITC (*p* < 0.001), camphor and menthol (*p* < 0.001), camphor and thymol (*p* < 0.001), as well as between AITC and menthol (*p* < 0.001) and AITC and thymol (*p* < 0.001) effects. No significant differences were observed between menthol and thymol effects (*p* = 0.946).

**Figure 1:**
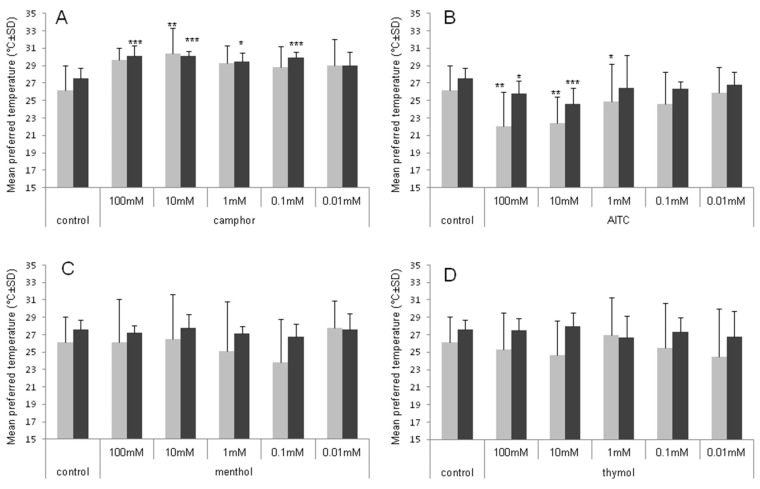
Thermal preference of the American cockroaches exposed to TRP ligands: Camphor (**A**), AITC (**B**), menthol (**C**) and thymol (**D**). Data are presented as the mean preferred temperature from the first 30 min (grey column) and from 1–23 h (black column) after exposure. Statistically significant differences in comparison to the control group are indicated as: * *p* < 0.05; ** *p* < 0.01; *** *p* < 0.001 (one-way analysis of variance (ANOVA) on ranks; *n* = 12).

Cockroaches revealed different thermopreference responses depending on the type of TRP ligand (supplementary material S1 Spreadsheet1). Significant increase in the preferred ambient temperature was observed after the application of camphor at concentration from 0.1 to 100 mM. At concentration of 100 mM and 10 mM of this substance, we observed a preference for about 2.5 °C higher ambient temperature compared to a control group (Figure 1).

Another thermopreference response was observed after application of AITC, thymol and menthol. Cockroaches revealed a tendency to stay in a cooler compartments of the thermal gradient. AITC induced preference for lower temperature in comparison to the control individuals, at higher concentrations: 100, 10 and 1 mM (in the last case only in the first 30 min of observation). TRPL inhibitors (menthol and thymol) had no significant effect on insect thermal behavior compared to the control.

### 2.2. Insects’ Head Temperature Changes in Response to TRP Agonists and Antagonists

The tested TRP ligands have been shown to induce changes in insects’ behavioral thermoregulation. Thus, in the next step of the study, we evaluated the influence of TRP ligands on cockroaches’ body temperature. In the preliminary experiments, the temperature of the head, thorax and abdomen was evaluated. The significant changes of temperature after ligands application were observed on the cockroach’s head; thus, we decided to present the results for the head temperature only.

**Figure 2:**
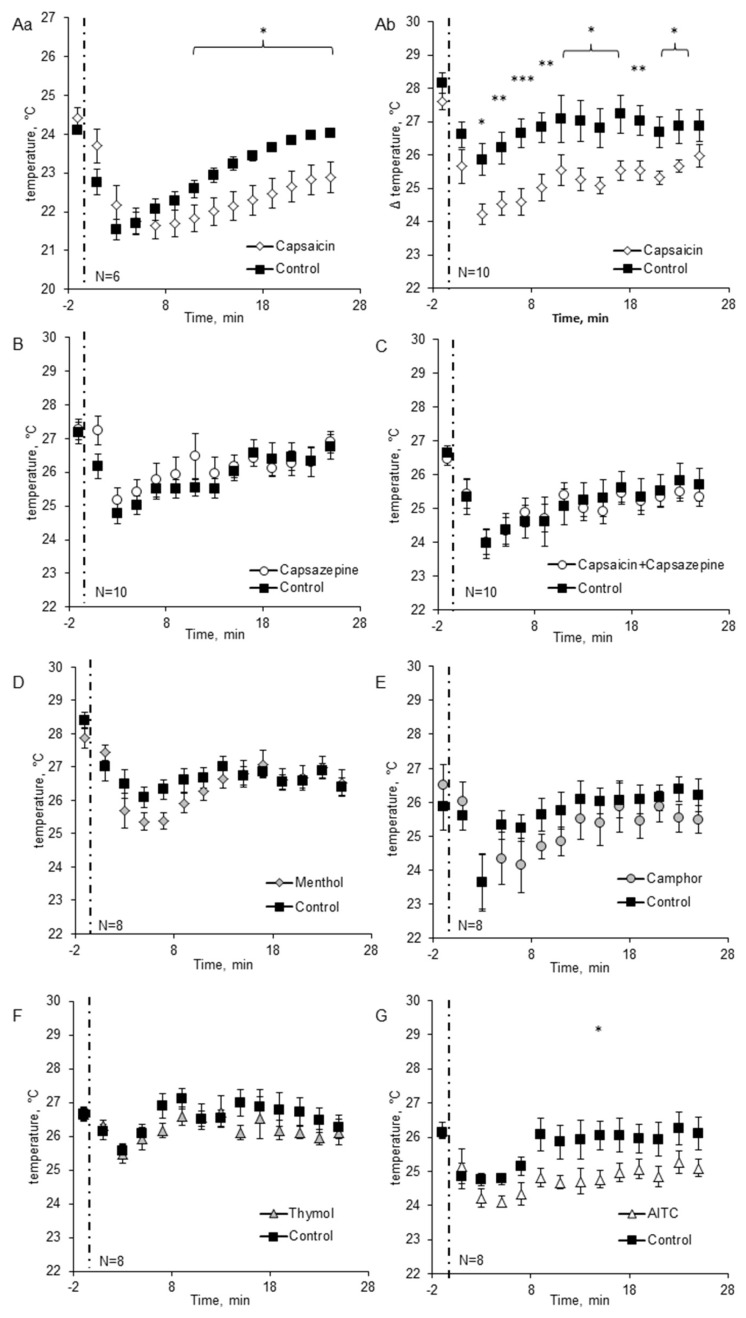
The temperature of the head of the cockroaches treated with TRP ligands (**A**). Changes of head temperature induced by 1 µM capsaicin measured using (**a**) thermocouple system and (**b**) thermal camera. Changes of the head temperature induced by 1 µM capsazepine (**B**), 1 µM capsazepine + 1 µM capsaicin (**C**), 0.1 mM menthol (**D**), 10 mM camphor (**E**), 1 mM thymol (**F**) and 0.1 mM AITC (**G**) measured with thermal camera. Time of application of tested substances is marked by dashed line. Data are presented as mean ± standard error of the mean (SEM). Note that the first data points on each graph refer to the moment just before the application. * indicates values significantly different vs. control group: * *p* < 0.05; ** *p* < 0.01; *** *p* < 0.001; least significant difference (LSD) test. The group sizes are indicated on the figure.

In the first set of experiments, the temperature of the insect’s head in control conditions and after capsaicin application was measured using thermocouple system. The average temperature of the head of the immobilized control insects was equal to 24.11 ± 0.09 °C; it was always slightly lower than the ambient temperature due to the evaporation of hemolymph from the placement of thermocouple. Application of alcohol or capsaicin on the insect’s thorax resulted in the decrease of head temperature because of fast alcohol evaporation that occurred up to 5 min; the biggest decrease (by 2.57 ± 0.31 °C) was observed 3 min after treatment (Figure 2Aa). In the 21th minute after application, the head temperature of control groups returned to the values from the beginning of the experiment and was equal to 23.84 ± 0.1 °C (*p* = 0.108 compared to the beginning of the experiment). After capsaicin application, the head temperature was the same as in the control group to the 9th minute, but the subsequent increase of temperature was much slower than after alcohol administration. As a result, the head temperature after capsaicin application was significantly lower than in control insects from the 11th minute to the end of the experiment (21.83 ± 0.34 °C vs. 22.59 ± 0.22 °C; *p* = 0.04) (Figure 2Aa). The decrease of temperature caused by the evaporation of hemolymph due to the placement of a thermocouple could overlap with the effect of alcohol and capsaicin; therefore, we decided to perform experiments using an alternative, non-invasive method—Measurement of temperature with thermal camera.

In the second set of experiments the temperature was measured using a thermal camera. In control conditions, the average temperature of the head of the immobilized insects, in all control experiments, was equal to 26.97 ± 0.17 °C. As in the first set of experiments, the use of all the solutions (alcohol in control groups) caused a decrease of temperature that occurred up to 5 min after administration; the biggest decrease (1.99 ± 0.2 °C) was observed 3 min after treatment (Figure 2). In the 15th minute after application, the head temperature of control groups almost returned to the values from the beginning of the experiments (26.48 ± 0.18 °C) and remained relatively stable to the end of the experiments. It is necessary to note that insects differed in motor activity (some of them tried to free themselves), and thus we observed some scatter of insects temperature between the experiments.

Application of TRP ligands changed the head temperature compared to control groups: treatment: d.f. = 15, F = 10.66, *p* < 0.001; time: d.f. = 1, F = 6.04, *p* = 0.014; treatment × time: d.f. = 15, F = 2.22, *p* = 0.004).

After capsaicin administration the significant change in cockroaches’ head temperature was observed to be similar to results obtained with thermocouple measurement system. The head temperature was lower than in the control group by 1.64–2.05 °C (*p* < 0.001); for example 7 min after capsaicin treatment. It was 24.53 ± 0.35 °C, whereas in control insects it was equal 26.64 ± 0.42 °C (*p* = 0.003). The differences were observed until about 25 min of registration (*p* = 0.04) (Figure 2Ab). Thus, the results of the experiments performed using both thermal camera and thermocouples were quite comparable. In both cases we could observe a significant decrease of the head temperature after alcohol and capsaicin application; moreover, in both cases the head temperature was significantly lower after capsaicin administration compared to the control group.

Capsazepine, the inhibitor of the TRPV1 receptor did not change the insects’ head temperature compared to the control group (*p* = 0.12) (Figure 2B), but applied together with capsaicin it abolished its effects (capsazepine with capsaicin vs. control *p* = 0.59) (Figure 2C).

Generally, the effects of other tested TPR ligands were minor (Figure 2D–G). The only significant effect (but lower than for capsaicin) was observed after AITC treatment. AITC exposure induced ‘cooling down’ of the cockroaches’ heads (Figure 2G); the difference between the control and the treated group was significant in the 15th minute after ligand application. The temperature of the cockroaches head was 24.77 ± 0.26 °C, while in the control group, the head temperature was equal to 26.04 ± 0.42 °C (*p* = 0.03).

Camphor, menthol and thymol had no effect on cockroach head temperature (Figure 2D–F) (camphor—*p* = 0.13; menthol—*p* = 0.17; thymol—*p* = 0.12) (supplementary material S1 Spreadsheet2).

## 3. Discussion

The investigation of the role of TRP receptors in insects’ thermoreception is continuously proceeding. We know a lot about *Drosophila’s* thermoreceptors but knowledge concerning other insect species is limited. Based on literature data, we decided to evaluate the effects of substances that have been proposed to act on insects TRP receptors [18,24,25,26,27]. We exposed adult individuals of the American cockroach to potential TRP ligands to compare their effects in a homogenous experimental group. The prevailing majority of available reports is based on in vitro experiments. However, some authors emphasize that there is some discrepancy between the native and heterologously expressed channels both in terms of channel properties as well as in the mechanism of channel activation [31]. Thus, the necessity to conduct behavioral experiments, in order to confirm in vitro results, is underlined (for example: [24]). The TRP receptors’ function depends on their insertion into a specific plasma membrane. Furthermore, TRP channels may interact with accessory proteins to form complexes, and the components of these complexes probably regulate gating and localization of TRP within specialized membrane domains [32].

Capsaicin induced the decrease of the head temperature of immobilized cockroaches. The influence of capsaicin on behavioral thermoregulation is already known [5]. However, according to our knowledge, the obtained results are quite new; until now, the effects of capsaicin and the antagonist of its receptor (capsazepine) have never been tested on insect physiological thermoregulation. In our study, we have shown that capsaicin induced cooling down of the cockroaches’ head temperature when insects did not have the possibility to change their thermal environment, whereas capsazepine had no effect: receptors were not activated and the head temperature was the same as in the control group, however capsazepine inhibited completely the effect of capsaicin.

We propose that in *Periplaneta americana* capsaicin activates heat receptors. However, it is still undiscovered which channel is responsible for the response to capsaicin—TRPV or other members of the TRP family. Based on current knowledge, we suggest that in the American cockroach, capsaicin affects some members of TRPA receptor family, which are known to be responsible for warm and heat detection in other species [15,16]. Some reports have shown the effects of capsaicin on insect behavioral thermoregulation, e.g., in *Tenebrio molitor* [23], *Rhodnius prolixus* [22] and the American cockroach [5,30]. These investigations also indicate that capsazepine acts as capsaicin antagonist, in a similar manner as in mammals. These data confirm our proposition that receptors sensitive to capsaicin are involved in cockroaches’ behavioral as well as physiological thermoregulation. Although, the behavioral thermoregulation in ectotherms plays a major role, there are also physiological mechanisms that lead to changes in body temperature: changes in heart beating or changes in mitochondrial capacities [33]. *Periplaneta americana* placed in high ambient temperature (40 °C) was able to keep the body temperature lower than the ambient one by almost 2 °C and in 45 °C lower by almost 3 °C. In such conditions the mouth part of the insect’s head had the lowest temperature which indicates special head cooling by evaporation [34]. However, such a mechanism can only play a short-term role because drying becomes a greater danger than heat.

Cockroaches’ thermoregulatory response to AITC was robust and homogenous, as in both types of experiments we obtained corresponding results. When the insects had an opportunity to choose ambient temperature they selected colder compartments in the thermal gradient; when they were immobilized, a decrease of the head temperature was observed. AITC activates Painless receptor in *Drosophila* that responds to noxious heat [25]. Our results suggest that cockroaches’ noxious heat receptor is also sensitive to AITC. On the other hand, this alkaloid was shown to activate mammalian cold receptor TRPA1 and probably also TRPV1 [35]. It was also demonstrated that AITC activates TRPV1 via interaction with the capsaicin-binding site [36]. Similarity in the response of the immobilized insects to capsaicin and to AITC observed in our experiment may indicate that both compounds act on the same noxious heat receptor in cockroaches.

Camphor was shown to activate *Drosophila* cold receptor TRPL [27]. Our results suggest that the same mechanism may be involved in the American cockroach. Activation of cold receptors would induce preference for warmer environment, as we have observed in our experiments. TRPL channels were found also in the American cockroach. It was shown that they play a major role in phototransduction, similarly to *Drosophila*, where light is detected by rhodopsin molecules and leads to activation of dTRP and dTRPL channels [37]. It could be expected that cockroach TRPL channel, apart from visible light detection, responds also to cold, similarly to *Drosophila*. Activation of the cold receptor should also induce an increase in body temperature. As was shown in rats, treatment with agonist of cold receptor (TRPM8) develops a rise in body temperature, as well as an increase in thermopreferendum [38]. Our results showed that body temperature of camphor-treated cockroaches, which had no opportunity to change their thermal environment, did not change. It is possible that physiological mechanisms for endogenous heat production were not efficient enough to increase insects’ body temperature. On the other hand, low temperatures are not as threatening for cockroaches (ectotherms) as intensive heat. It has been shown that cockroaches’ body temperature equalled to ambient temperature in the rest conditions [39]. They can survive even at temperatures close to 0 (personal observation). Moreover, it is also known, that ectotherms have a longer lifespan at lower ambient temperatures [33]. Contrary, *Periplaneta americana* exposed to high ambient temperatures or during flight may lose heat due to evaporative cooling [40]. Activation of thermoregulatory processes may also have an effect on heat detection after exposure to capsaicin or AITC. Camphor was also shown to inhibit the heat-induced activation of Painless receptor in *Drosophila* [24]. We previously demonstrated that repeated treatment with camphor increases the latency to escape from noxious heat in the American cockroach [7]. Results obtained in the present study correspond well with these data—Insects after camphor treatment found the optimal environment for the control insects as too cold and chose higher temperatures. It is interesting to observe that camphor did not cause a dose-dependent effect. Perhaps it is possible that the maximum effect was already reached with one of the lowest doses (0.1 mM) used in the study, so no further effect of the increased concentration was observed. This is called the ‘ceiling effect’. This effect was shown for several substances, for example, buprenorphine, which reveals a ceiling effect for respiratory depression [41]. Paracetamol was shown to have a ceiling effect in postoperative pain [42]. Moreover, some studies suggest that AITC at high doses also reveals a ceiling effect. Andersen et al. showed that in humans, AITC produces a spontaneous burning pain, mechanical and heat hyperalgesia with a ceiling effect from 50% to 90% concentration [43].

Contrary to the effect of camphor, menthol and thymol are known to inhibit *Drosophila’s* cold receptor TRPL. Our results confirm the opposite effect of camphor and the effect of menthol or thymol on the cockroaches’ thermopreference (significant difference between camphor and menthol or thymol effect). We also observed only minor changes in the body temperature after exposure to menthol and thymol (no significant effect). In rats, pharmacological blockade of cold receptor decreased deep body temperature [38]. Therefore, our results did not confirm the role of these two alkaloids as TRPL inhibitors in the American cockroach.

To conclude, the thermoregulatory responses to the TRP ligands in the American cockroach indicate involvement of TRP receptors in cockroaches’ thermosensation.

Based on the obtained results and our previous experiments [5,7], we suggest that in the American cockroach:

1/ capsaicin and allyl isothiocyanate (AITC) act on heat receptor, probably some member of TRPA family

2/ capsazepine acts as antagonist to capsaicin

3/ TRPL receptors could act as cold receptor and they are activated by camphor

## 4. Materials and Methods

Experiments were performed on the adult individuals of the American cockroach, *Periplaneta americana* L. Insects were reared in plastic containers at ~26 °C and 12:12 LD.

### 4.1. Substances Tested

Capsaicin, capsazepine, camphor, menthol, thymol, and allyl isothiocyanate were obtained from Sigma-Aldrich (St. Louis, MO, USA) and dissolved in ethyl alcohol (96%) to obtain the desired solutions. The substances examined were applied topically (5 µL) on the thorax. The control group was treated only with alcohol.

### 4.2. Behavioral Assays

Behavioral thermopreference of cockroaches was determined in the thermal gradient system. The apparatus consisted of a long and narrow aluminum trough (600 × 50 mm) with water chambers on both ends. One water chamber was filled with cold water pumped from a cryostat (Polyscience 9106, Niles, IL, USA), while the second one with hot water pumped from thermostat (Polyscience 8006, Niles, IL, USA), which allowed to create a temperature gradient inside the trough, ranging from 10 °C to 40 °C. The change of temperature along the trough was approximately 0.5 °C/10 mm. The trough was divided into 20 compartments of equal length in which ambient temperature was measured with a thermocouple before each experiment.

An individual cockroach, directly after exposure to the tested substances, was placed in the middle of the trough at the temperature of 23.8 °C ± 0.1 °C. Its thermal preferences were recorded for 24 h with a camera (Sony FDR AX33, Sony Electronics Inc., San Diego, CA, USA) and data were saved on a computer disc. All experiments were performed in the 12 h cycles of light and dark. Each experiment started at 9 am. The preferred temperatures were determined based on the position of the examined cockroach in the thermal gradient; for each tested substance 12 individuals were examined. The thermal preferences of each individual were established from data obtained every 10 min in the first hour of the experiment and then every 2 h until the end of the experiment. Each cockroach’s position was ascribed to specific ambient temperature.

### 4.3. Body Temperature Measurement at Constant Ambient Temperature

We also evaluated the ability of the cockroaches to thermoregulation, which could be related to endogenous processes. For this purpose, we measured the temperature in the immobilized insects in constant thermal conditions −24–25 °C. Experiments were performed in the open styrofoam chamber (200 × 300 mm) and animals were attached to 35 mm plastic Petri dishes by double-sided tape placed on the dorsal side (upside down position). After adaptation period (time sufficient to stabilize the insect’s body temperature), tested substances, dissolved in ethanol to desired concentrations, were applied using Hamilton syringe on the insect’s thorax. The control group for each ligand was treated with alcohol of the same volume (5 µL). The temperature of the insect was estimated using two methods: (1) registration with thermocouple measurement system [44] and (2) registration with thermal camera.

(1) Thermocouple measurement system. The temperature of the cockroach’s head was measured using a copper-constantan thermocouple made from 0.05 mm-diameter wires. The thermocouple was implanted into the head just under the cuticle and fixed to the cuticle with TAKI Wax 20 min before the experiment. Since the thermocouples are differential devices rather than absolute temperature measurement devices [45], the temperature of the insect’s head was measured as a difference to the chamber temperature with an accuracy 0.2 °C using a Line Recorder TZ 4620 (Laboratorni Pristroje Praha, Prague, Czech Republic). In parallel, the chamber temperature was measured every 2 min and these values were used to express insect head temperature in absolute values. The changes in temperature referred to the changes in voltage on the thermocouples endings (for Cooper-Constantan thermocouple 40 µV refers to 1 °C). The placement of the thermocouple in insect head was associated with a slight damage of the cuticle through which hemolymph could evaporate and what could cause the decrease of the measured temperature.

(2) Thermal camera. After 10 min of adaptation, the insects’ temperature was recorded every 2 min over 25 min using Flir One Thermal Camera (Flir Systems, Wilsonville, OR, USA) (8–14 µm spectral range; 100 mK thermal sensitivity; 4800 pixels thermal image resolution). The camera was placed 30 cm from the cockroaches at an angle of 45°, which let us avoid infrared reflection. Since living organisms are characterized by emissivity equal to 0.96–1.00, the emissivity on thermal camera was set to 1.00 (matt surface). Data were stored on a mobile device and further analyzed with dedicated software, Flir Tools (3.9.1, Flir Systems, Wilsonville, OR, USA). The absolute insects’ body temperature was estimated. Thermogram images (supplementary material S1 Image) allowed us to identify the temperature for specific regions of the insects’ bodies (head, thorax and abdomen). In all experiments, the temperatures of two treated individuals were recorded simultaneously with the temperatures of two control insects.

### 4.4. Data Analysis

To analyze the effect of the substances and their concentrations (camphor, AITC, menthol and thymol) on cockroaches’ thermal preferences, we applied a generalized linear mixed model (GLMM). We included measurement time (hours of the test) as a continuous variable and replicate as a random factor (to avoid pseudoreplications). Thermal preference was used as a dependent variable while substance (camphor, AITC, menthol and thymol) and their concentrations (100, 10, 1, 0.1, 0.01 mM) as fixed factors. Significant terms were further explored using least significant difference (LSD) post hoc tests. Differences between the control and the treatments groups were analyzed separately for each concentration of the tested substance using one-way analysis of variance (ANOVA) on ranks. Each analysis was followed by multiple comparisons using Dunn’s post hoc test.

The treatment effect on the insects’ head temperature was evaluated with a one-way generalized linear mixed model (GLMM). We included measurement time (minutes of the test) as a continuous variable and replicate as a random factor (to avoid pseudoreplications). Head temperature was used as a dependent variable while substance (camphor, AITC, menthol and thymol) as main factor. Each analysis was followed by multiple comparisons using Fisher’s LSD post hoc test.

All analyses were conducted in IBM SPSS 25 Statistics software (IBM Corporation, Armonk, NY, USA). The differences were considered as statistically important when *p* < 0.05.

## Figures and Tables

**Table 1:** Insect thermo-TRP and their chemical agonists or antagonists.

Type of Receptor Subfamily	Name	Ligands that Affect Receptors Activity	Literature
Warmth receptors TRPA	Painless (>42 °C)	allyl isothiocyanate (activation) camphor (inhibition)	[15,24,25]
	Pyrexia (>35 °C)	?	[15]
	dTRPA1 (25–27 °C)	allyl isothiocyanate (activation)	[26]
	HsTRPA (Hymenoptera specific TRPA) (>36 °C)	camphor (activation), menthol (inhibition)	[18]
	TRPA5	?	[17]
Cold receptors	TRP	camphor (activation), thymol and menthol (inhibition)	[27]
TRPC	TRPL (10–20 °C)		
TRPP	Brivido 1, −2, −3 (12–15 °C)	?	[19]
TRPV	Inactive (10–20 °C)	?	[20]
NOMPC	Nompc	?	[21]
TRPM	Trpm		
TRPC	Pkd2 (<10 °C)		

?—ligands not known.

**Table 2:** Effect of different ligands on invertebrates’ TRP channels.

Concentration	Effect	Species	Literature
**Camphor**			
3 mM	proboscis extension reflex reduction	honey bee	[18]
4.3 mM (EC_50_)	AmHsTRPA activation	honey bee	[18]
3 mM	heat-induced activation of Painless inhibition	*Drosophila*	[24]
10 mM	TRPL activation	*Drosophila*	[27]
15 mM (repeated doses)	desensitization to heat	*Periplaneta americana*	[7]
**Allyl isothiocyanate**			
50 µM	HarmTRPA1 activation	*Helicoverpa armigera*	[28]
1 mM	AmHsTRPA activation proboscis extension reflex reduction	honey bee	[18]
2 mM	proboscis extension reflex reduction through Painless	*Drosophila*	[25]
2 mM	proboscis extension reflex reduction through dTRPA1	*Drosophila*	[26]
**Menthol**			
100 nM (EC_50_)	AmHsTRPA inhibition	honey bee	[18]
0.5 mM	increased fraction of bees in warmer regions	honey bee	[18]
1.8 mM	TRPL inhibition	*Drosophila*	[27]
**Thymol**			
1 mM	TRPL inhibition	*Drosophila*	[27]
**Capsazepine**			
0.1 µM	preference for warmer regions	*Periplaneta americana*	[5]
0.1 µM (repeated doses)	desensitization to heat	*Periplaneta americana*	[7]
0.1 µM 100 µM	preference for warmer regions preference for cooler regions	mealworm *Tenebrio molitor* larvae	[23]
26 µM	preference for cooler regions (1–1.5 °C); increase in proboscis extension reflex	*Rhodnius prolixus*	[22]
1 mg/kg	increase in heat-evoked latencies	snail *Megalobulimus abbreviatus*	[8]
18 µM	block TRPM8 response to menthol	HEK293 cells	[29]
**Capsaicin**			
0.1 µM	preference for cooler environments	*Periplaneta americana*	[5]
100 µM	preference for warmer environments	*Periplaneta americana*	[30]
0.1 µM and 100 µM (repeated doses)	desensitization to heat	*Periplaneta americana*	[7]
0.1 µM 100 µM	preference for cooler environments	mealworm *Tenebrio molitor* larvae	[23]
3–12 µM	positive preference for capsaicin, also in painless mutants	*Drosophila*	[25]
0.34 mM	preference for higher temperatures (2.6 °C); less responsiveness to heat	*Rhodnius prolixus*	[22]
0.5%	decreased latency for withdrawal behavior from 50 °C	snail *Megalobulimus abbreviatus*	[8]
0.5 mM	elicited nocifensive behavioral responses	medicinal leech	[9]

## Supplementary Materials

The following are available online, S1 spreadsheet1: Raw data for temperatures preferred by the examined cockroaches in the thermal gradient, S1 Spreadsheet2: Raw data for head temperature measurements, S1 Image: Cockroach body temperature after treatment with capsaicin (1 µM) and thymol (1 mM) visualized with thermal camera. Note that capsaicin-treated cockroach’s body is colder than control or thymol-treated ones.
