# Supplementary figures and images for: Effect of Capsaicin and Other Thermo-TRP Agonists on Thermoregulatory Processes in the American Cockroach
Source: Molecules. 2018 Dec 18;23(12):3360. doi: 10.3390/molecules23123360 (PMC6321544; doi:10.3390/molecules23123360)

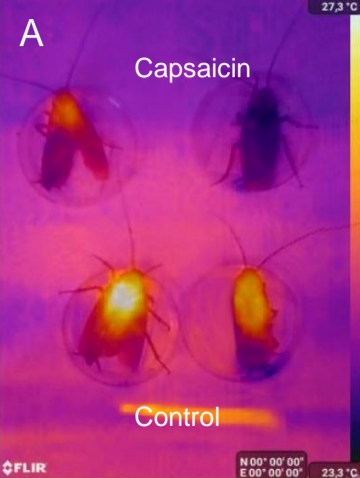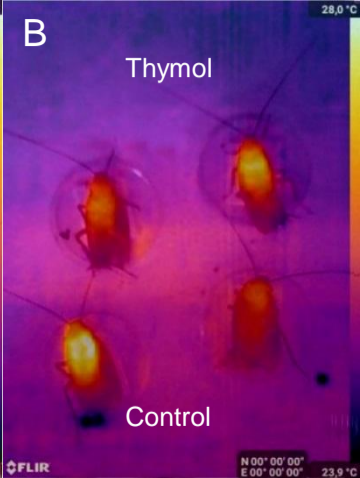

Supplement: Supplementary file 1 [file molecules-23-03360-s001.zip › S1 image.pdf]
